# Utilization of Outpatient Mental Health Services During and After The COVID-19 Pandemic

**DOI:** 10.23889/ijpds.v9i5.2622

**Published:** 2024-09-18

**Authors:** Michelle Hayek, Sulki Park, Mark Lawley, Michelle Bovin 3, Hye-Chung Kum

**Affiliations:** 1 Texas A&M University; 2 The University of Texas Health Science Center at Houston; 3 VA Boston Healthcare System; 4 Boston University Chobanian & Avedisian School of Medicine

## Objective and Approach

 This study examines the impact of COVID-19 on outpatient mental health (OP-MH) service use and assesses the role of telehealth on OP-MH service patterns. Using the 5% national sample of Medicare beneficiaries, the intervention group was defined as those newly diagnosed with a MH condition in 2019 (no MH diagnosis 2 years prior) with a 2-year study period of 2020-2021. The comparison group included non-overlapping individuals newly diagnosed in 2017 with a study period of 2018-2019. The OP utilization patterns were examined over the 2 years post-diagnosis and compared across MH diagnostic categories, including schizophrenia, bipolar, depression, anxiety, trauma-related, and substance-use disorders. 

## Results

The intervention group observed a significant reduction in MH utilization in March-April 2020 compared to the matched sample. While patients with schizophrenia and bipolar disorders saw a return to normal patterns by May 2020, those with depression, anxiety, trauma-related, and substance-use disorders continued to exhibit significant reductions in MH utilization post-COVID-19. The intervention group demonstrated a significant increase in telehealth usage, transitioning from 1% pre-COVID-19 to a peak of ~60% during the pandemic. This trend gradually decreased, reaching an average of 30% by the end of 2021 across all conditions.

## Conclusions and Implications

The intervention group observed a significant reduction in MH utilization in March-April 2020 compared to the matched sample. While patients with schizophrenia and bipolar disorders saw a return to normal patterns by May 2020, those with depression, anxiety, trauma-related, and substance-use disorders continued to exhibit significant reductions in MH utilization post-COVID-19. The intervention group demonstrated a significant increase in telehealth usage, transitioning from 1% pre-COVID-19 to a peak of ~60% during the pandemic. This trend gradually decreased, reaching an average of 30% by the end of 2021 across all conditions.

